# Supplementary material for: Recent changes to Arctic river discharge
Source: Nat Commun. 2021 Nov 25;12:6917. doi: 10.1038/s41467-021-27228-1 (PMC8617260; doi:10.1038/s41467-021-27228-1)
Supplement: Supplementary file 1 — Supplementary Information [file 41467_2021_27228_MOESM1_ESM.pdf]

Supplementary Information for

Recent changes to Arctic river discharge

Dongmei Feng[1*], Colin J. Gleason[1], Peirong Lin[2], Xiao Yang[3], Ming Pan[2,4], Yuta Ishitsuka[1]

[1]Department of Civil and Environmental Engineering, University of Massachusetts, Amherst, MA, USA.

[2]Department of Civil and Environmental Engineering, Princeton University, NJ, USA

[3]Department of Earth, Marine and Environmental Sciences, University of North Carolina at Chapel Hill, NC, USA.

[4]Center for Western Weather and Water Extremes, Scripps Institution of Oceanography, University of California San Diego, La Jolla, California, USA

[*]Corresponding author: Dongmei Feng (dongmeifeng@umass.edu)

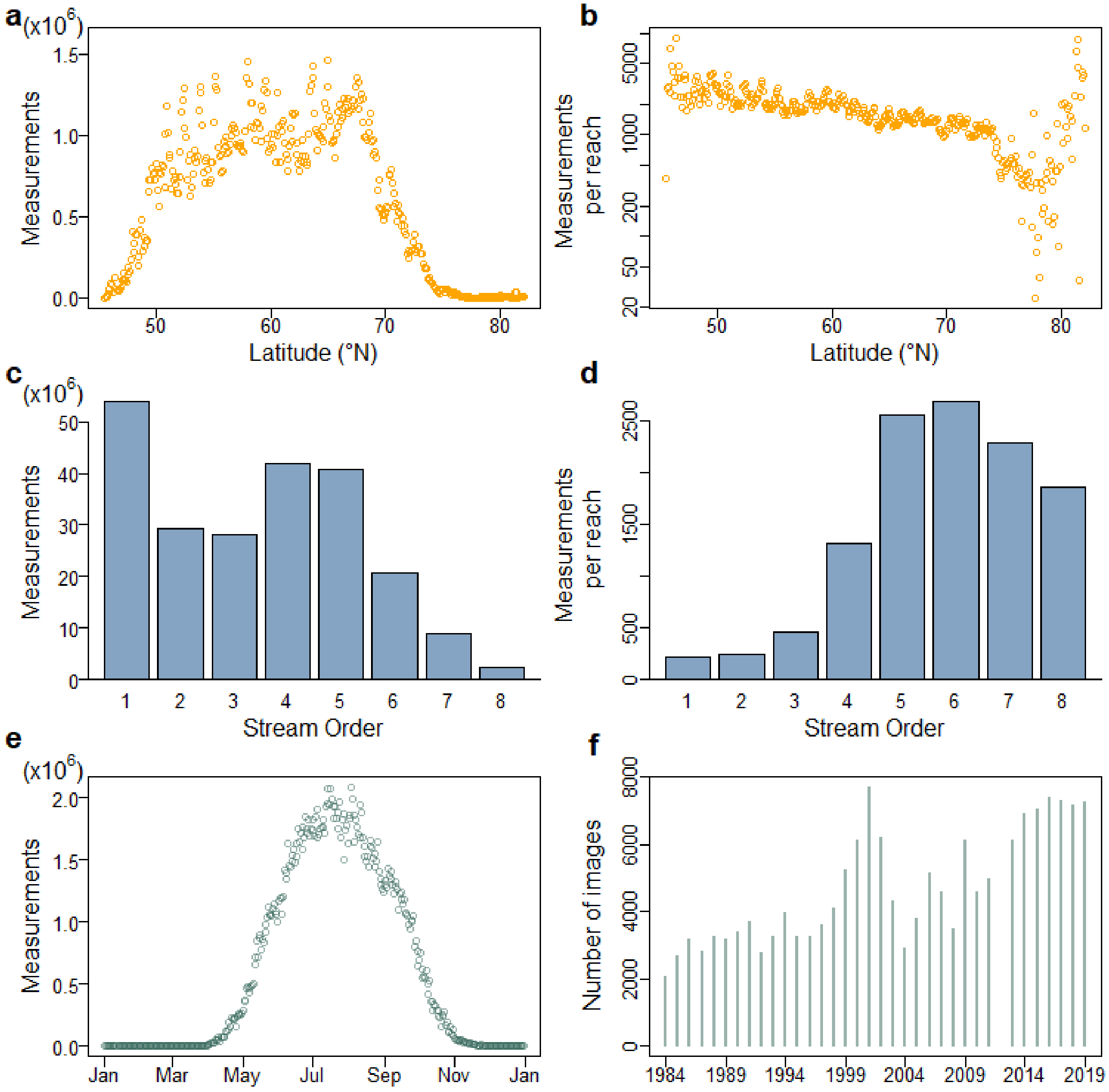

Supplementary Figure 1: Distribution of remote sensing river width measurements across space, stream order, and time. ***a****: Total number of river width measurements per latitude;* ***b****: Total number of river width measurements per reach per latitude (bin size 0.1 degree);* ***c****: Total number of river width measurements across stream orders;* ***d****: As* ***c*** *but normalized to the number of reaches per order;* ***e****: Total number of river width measurements across months;* ***f****: The number of images for width measurements across years in the study time domain.*

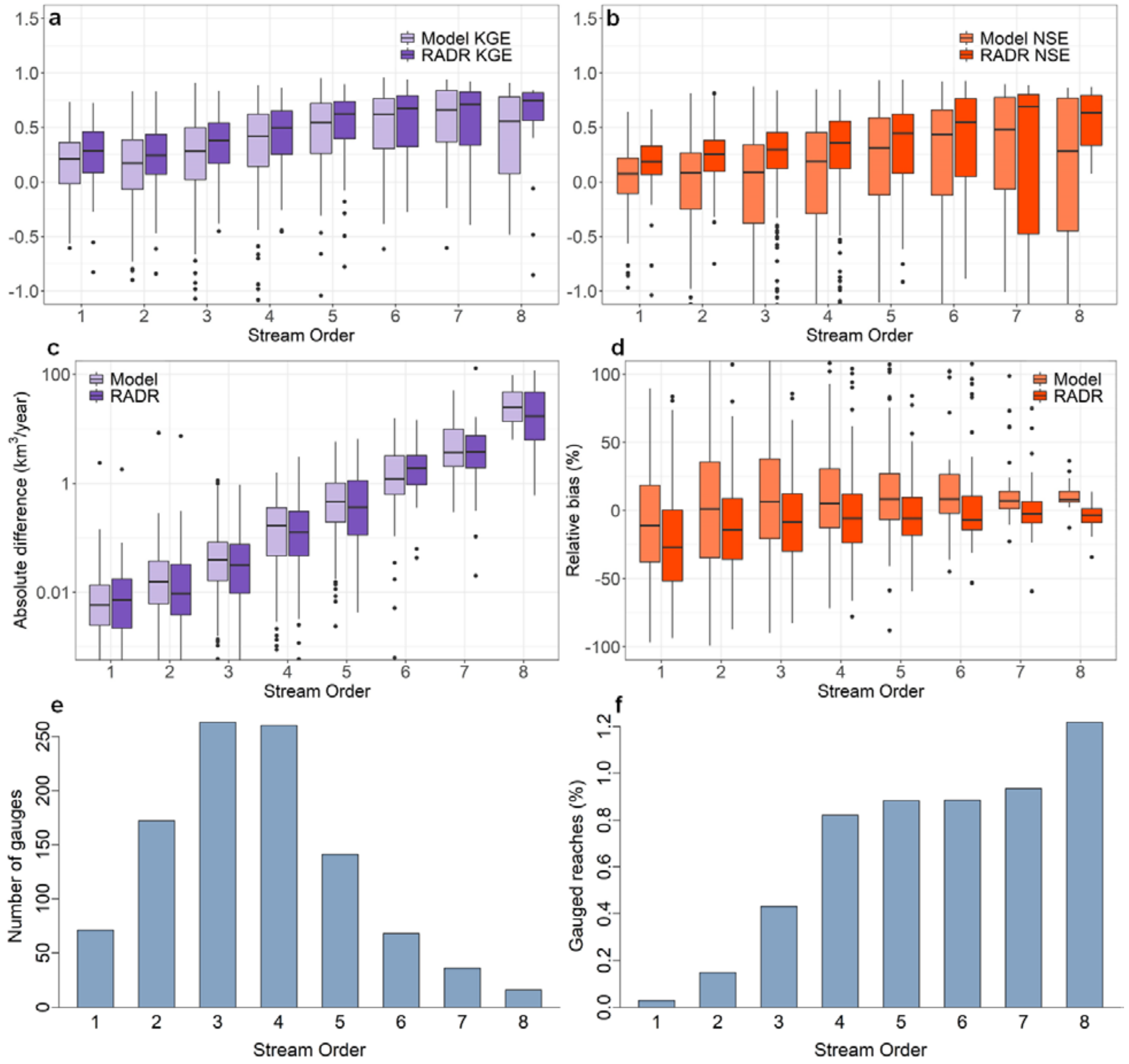

Supplementary Figure 2: Daily error metrics of RADR and baseline model against gauge data across river sizes (quantified by stream orders). ***a:*** *KGE****;*** ***b:*** *NSE;* ***c****: Absolute difference ($|Q_{sim} - Q_{obs}|$, km³/yr);* ***d****: Relative bias ($(Q_{sim} - Q_{obs})/\ Q_{obs} \times 100$, %);* ***e****: Number of daily gauges per stream order;* ***f:*** *The fraction (%) of gauged reaches in each stream order. In each boxplot, the highest and lowest points of the whiskers are the maximum and minimum KGE or NSE (excluding the outliers); the top and bottom of the box indicate 75% and 25% values of KGE or NSE; the dark black line in the box is the median of KGE or NSE; black dots are outliers, which are defined as data points that are located outside whiskers of the boxplot (i.e., outside 1.5 times the interquartile range above the upper quartile and below the lower quartile). This figure suggests that smaller rivers show the largest improvement from RADR, but these smallest rivers are by far the least gauged rivers.*

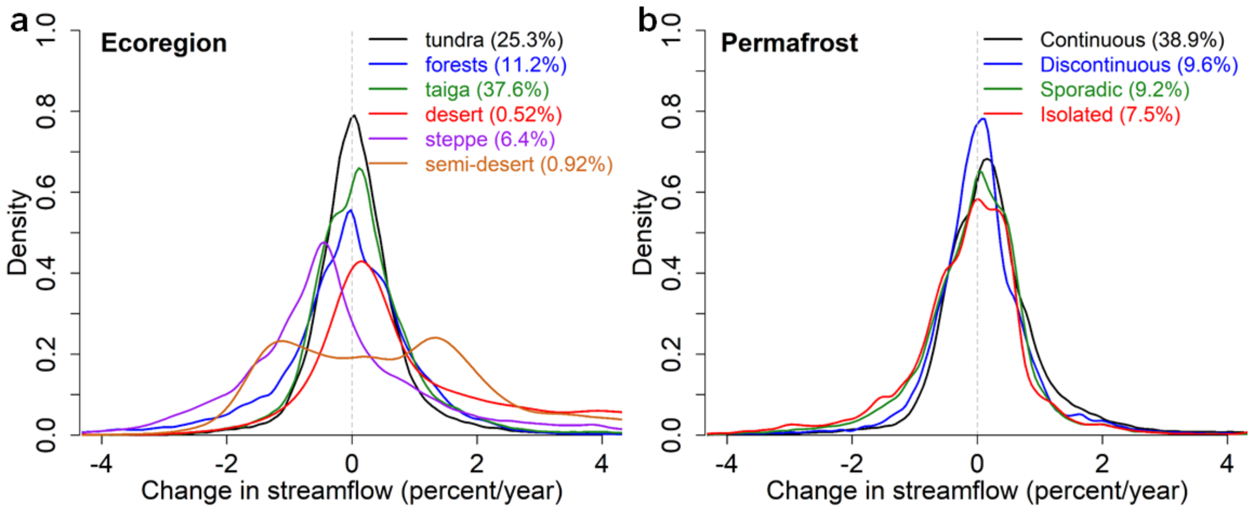

Supplementary Figure 3: ***a:*** *Probability Density Functions (PDFs) of streamflow changes for six major ecoregions of the pan-Arctic show regional variations; numbers in the legend indicate the percentage of the total land area of each ecoregion, calculated as the total area of each classification divided by the total land surface area of the pan-Arctic region. Streamflow changes are attrubited to the land classification at each reach, which could drain an area of mixed ecoregion/permafrost type.* ***b:*** *As* ***a****, but across four major permafrost categories.*

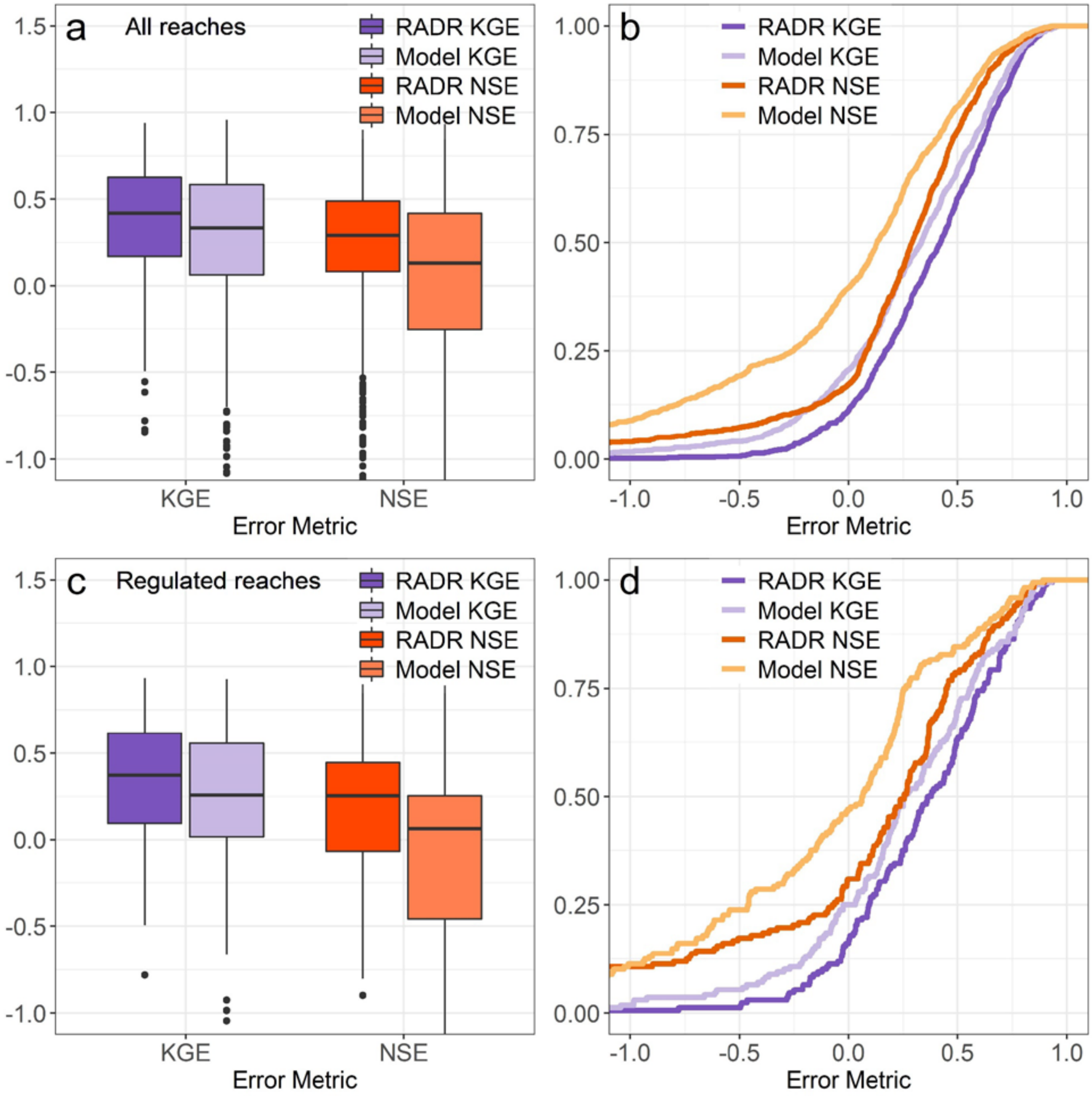

Supplementary Figure 4: ***a****: Error metrics (NSE and KGE) of RADR and the baseline model simulation validated at 1,079 validation gauges at daily time scales; The median NSE and KGE of RADR increase by 0.16 and 0.09, respectively, compared to those of the baseline model simulation; similar improvements can be obtained when only reaches with at least 30 years of gauge daily data are evaluated;* ***b****: Cumulative distribution function of NSE and KGE for RADR and baseline model for all gauged reaches;* ***c****: As* ***a****, but for regulated reaches; The median NSE and KGE of RADR increase by 0.19 and 0.11, respectively, compared to those of the baseline model simulation;* ***d****: As* ***b****, but for regulated reaches. In each boxplot, the highest and lowest points of the whiskers are the maximum and minimum KGE or NSE (excluding the outliers); the top and bottom of the box indicate 75% and 25% values of KGE or NSE; the dark black line in the box is the median of KGE or NSE; black dots are outliers. See Methods for the definition of*

the regulated reach. This figure suggests that RADR improves discharge estimates across the entire error distribution compared to baseline model simulations.

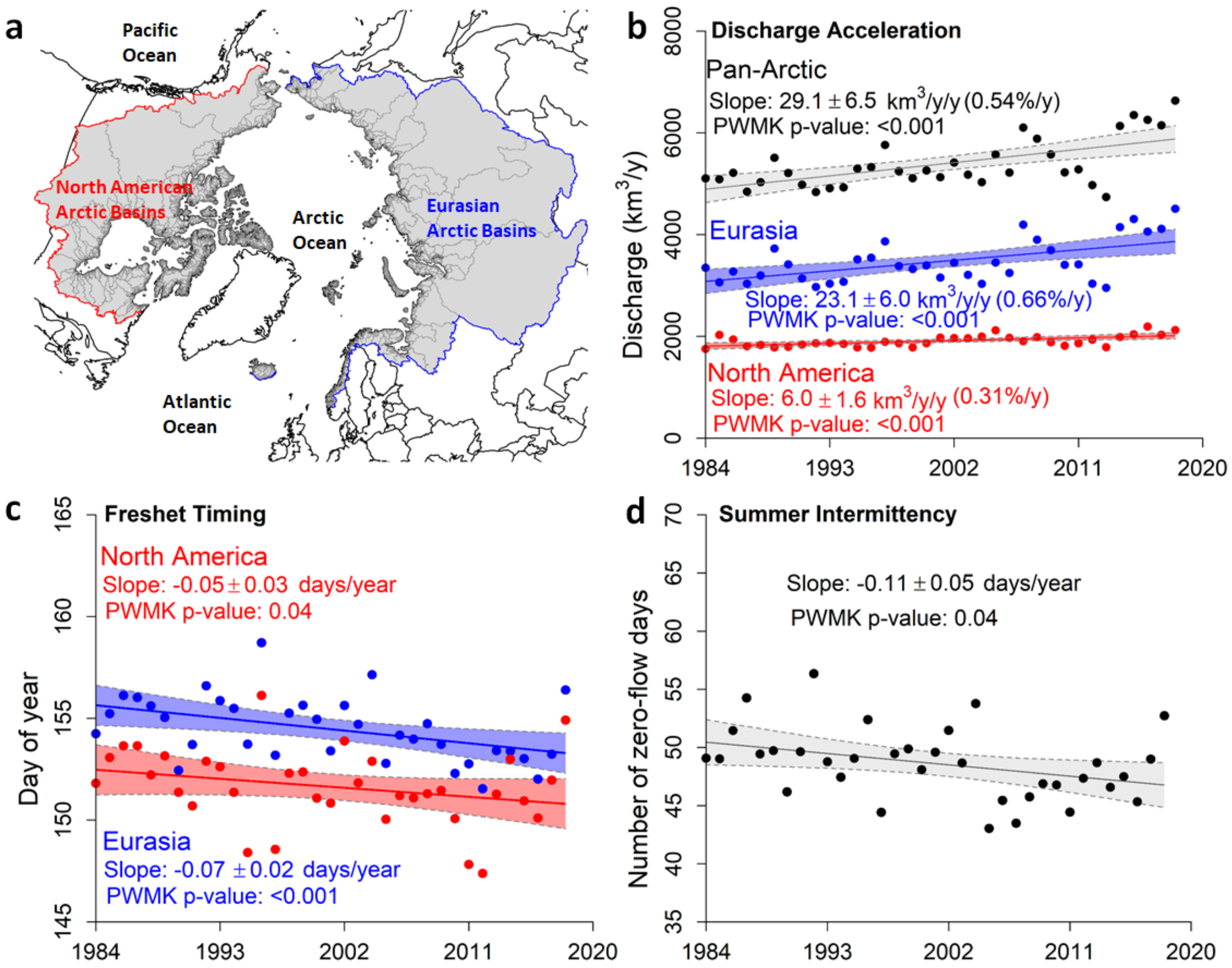

Supplementary Figure 5: Same as Figure 1 but based only on the baseline model simulation result. ***a:*** *Map showing the pan-Arctic (grey shaded), North American (outlined in red) and Eurasian (outlined in blue) basins;* ***b:*** *Water export from rivers in the pan-Arctic, Eurasia, and North America shows significant increasing trends;* ***c****: Changes in* ***t****he temporal centroid of spring freshet (TCSF) for North American and Eurasian rivers;* ***d:*** *Changes in the number of zero-flow days (ZFD) during the open-water period (Apr-Nov) for streams prone to intermittency. The shade in* ***b****,* ***c****, and* ***d*** *indicates the 95% confidence interval. Comparing this figure with Figure 1 gives the difference between RADR and the baseline model in terms of total water export, freshet timing, and river intermittency. See Methods for the definition of the baseline model simulation. PWMK refers to the pre-whitening Mann-Kendall test for trend significance.*

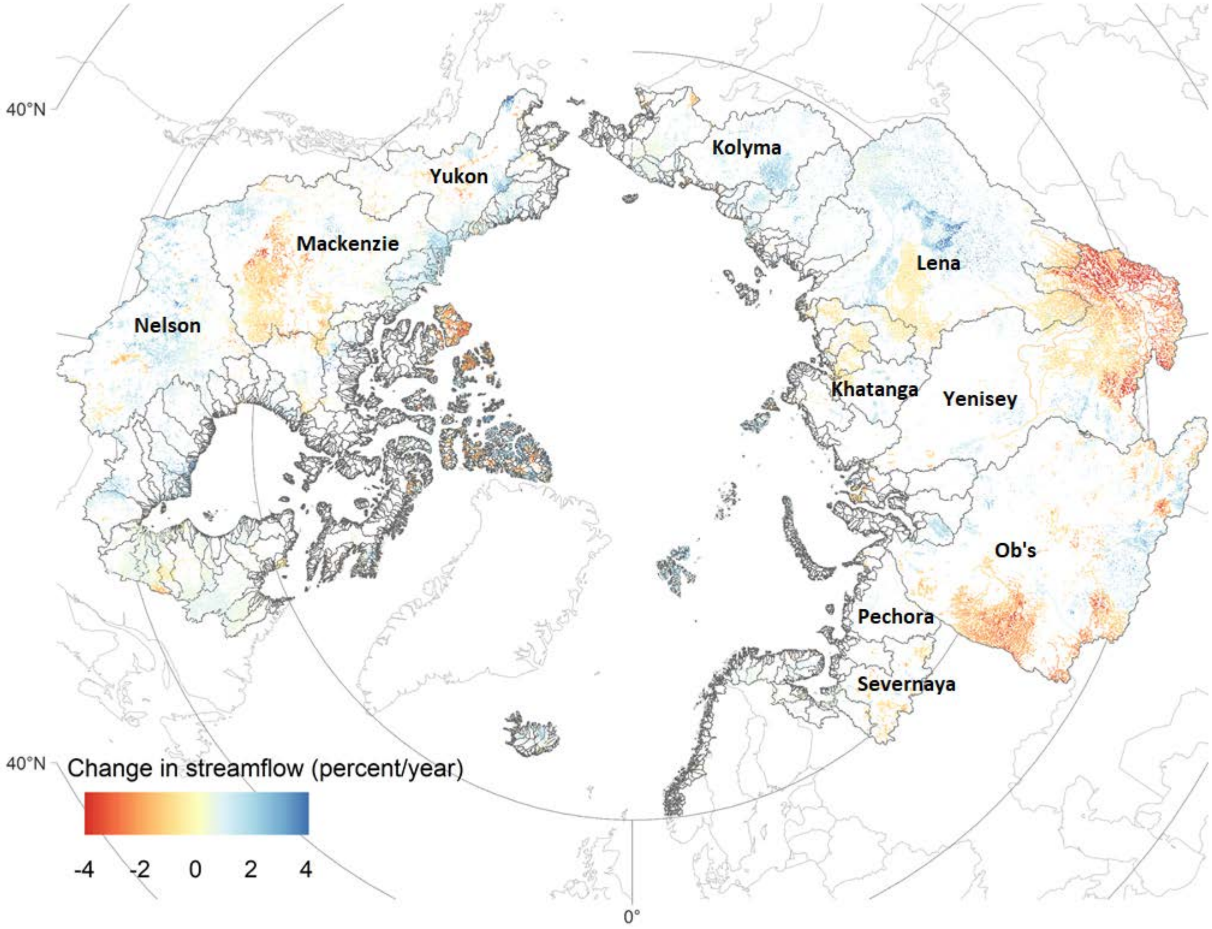

Supplementary Figure 6: *Same as Figure 2 but based only on baseline model simulations. Only rivers with statistically significant discharge trends are mapped (percent/year, p-value<0.05). Comparing this figure with Figure 2 gives the differences between RADR and the baseline model in terms of river discharge changes across space. See Methods for the definition of the baseline model simulation.*

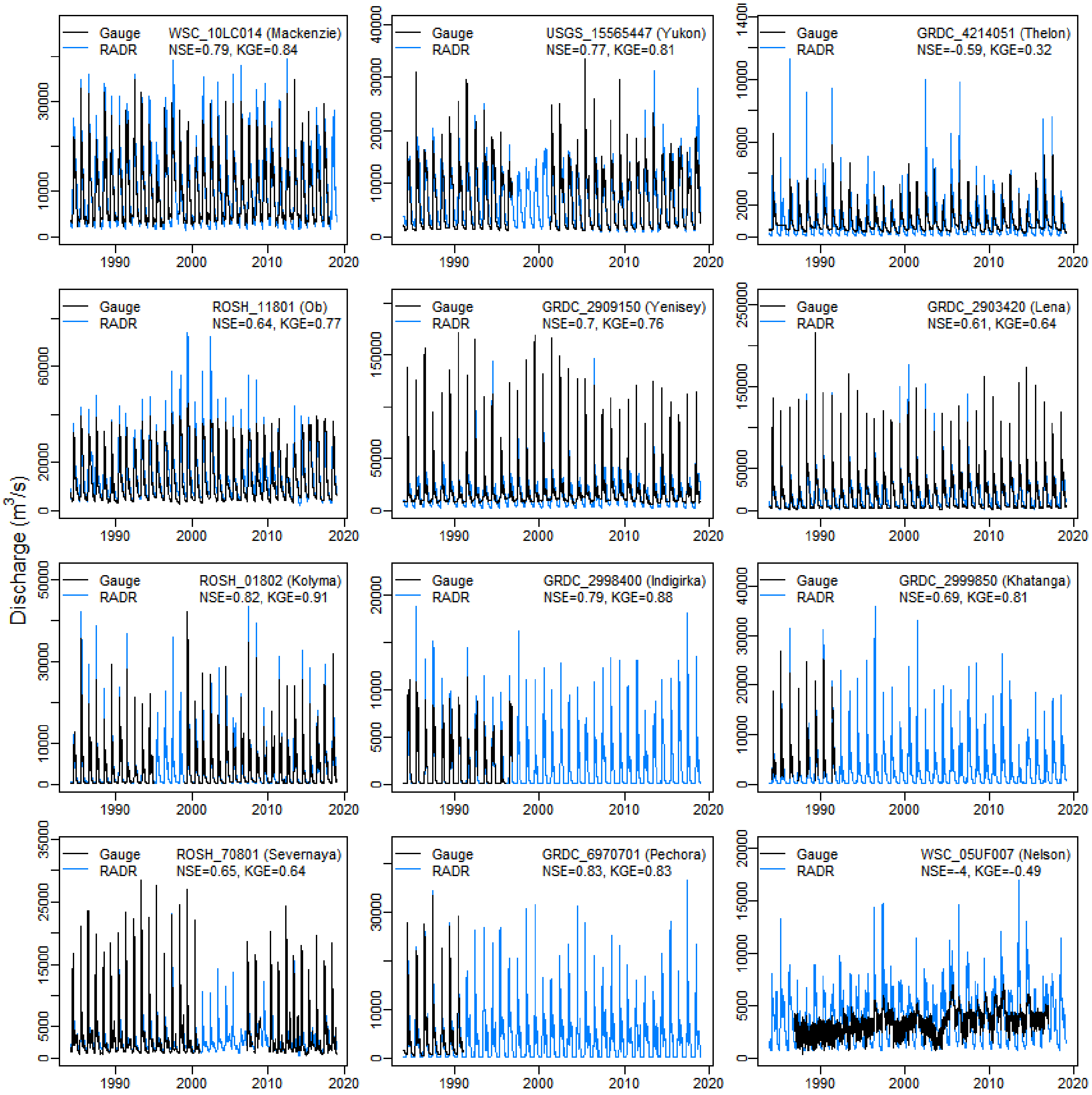

Supplementary Figure 7: Hydrographs of RADR compared with gauge observations for 24 large basins in the pan-Arctic region. Error metrics (KGE and NSE), station IDs, and river names are shown in the figure. Station IDs are labeled in the format of 'Agency_stationNumber.' WSC: Water Survey of Canada; GRDC: Global Runoff Dataset Center; ROSH: Roshydromet; and USGS: U.S. Geological Survey.

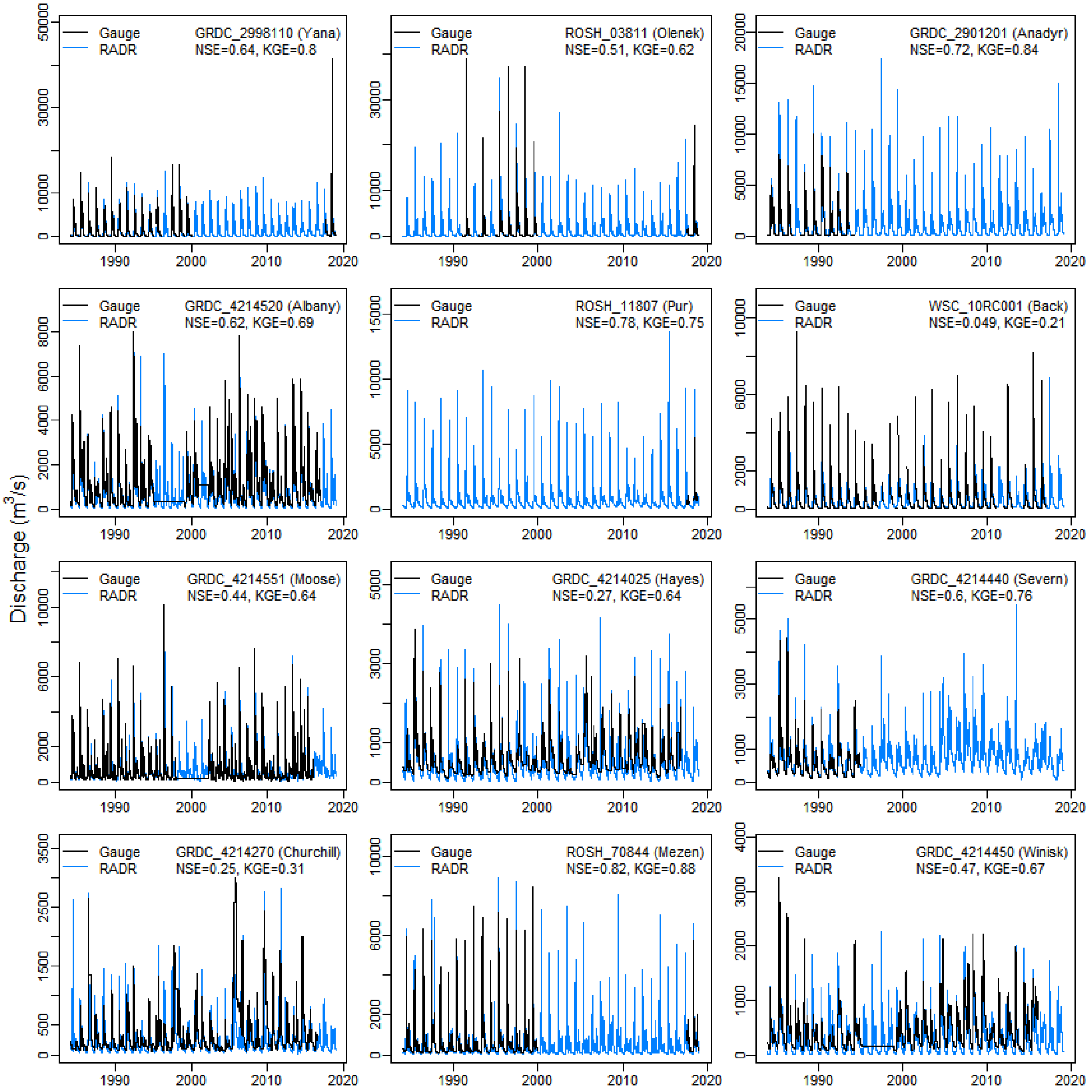

Supplementary Figure 7 (continued): Hydrographs of RADR compared with gauge observations for 24 large basins in the pan-Arctic region. Error metrics (KGE and NSE), station IDs, and river names are shown in the figure. Station IDs are labeled in the format of 'Agency_stationNumber.' WSC: Water Survey of Canada; GRDC: Global Runoff Dataset Center; ROSH: Roshydromet; and USGS: U.S. Geological Survey.

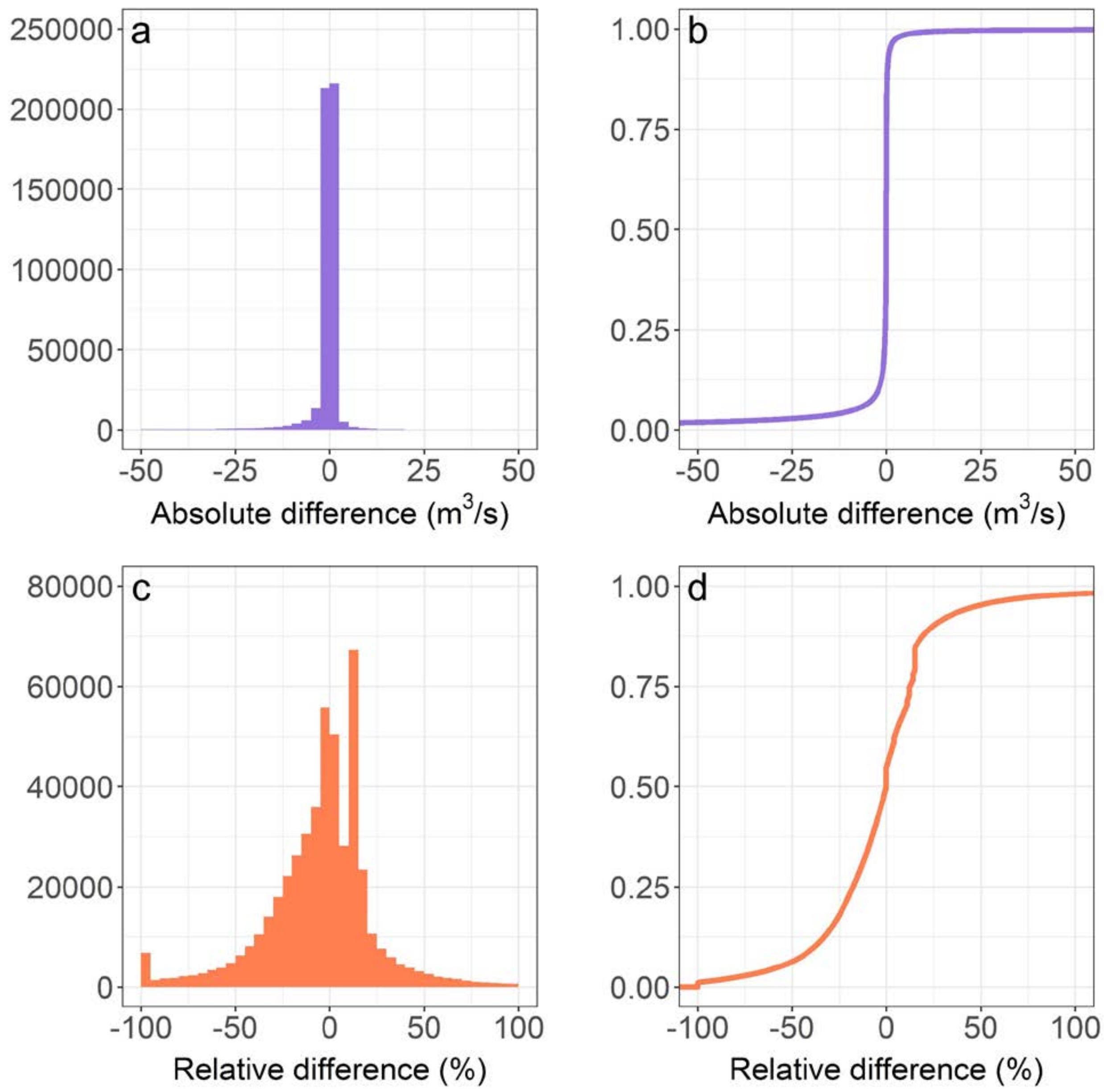

Supplementary Figure 8: Difference between RADR and baseline model simulations. ***a****: Histogram of absolute difference (m^3/s) between RADR and baseline model;* ***b****: Cumulative distribution function of the absolute difference (m^3/s) between RADR and baseline model.* ***c****: Histogram of relative difference (%) between RADR and baseline model;* ***d****: Cumulative distribution function of the relative difference (%) between RADR and baseline model; Positive (negative) values indicate RADR is higher (lower) than the baseline model. This figure suggests that the large relative differences mapped in Figure 3 are found almost exclusively on smaller rivers.*

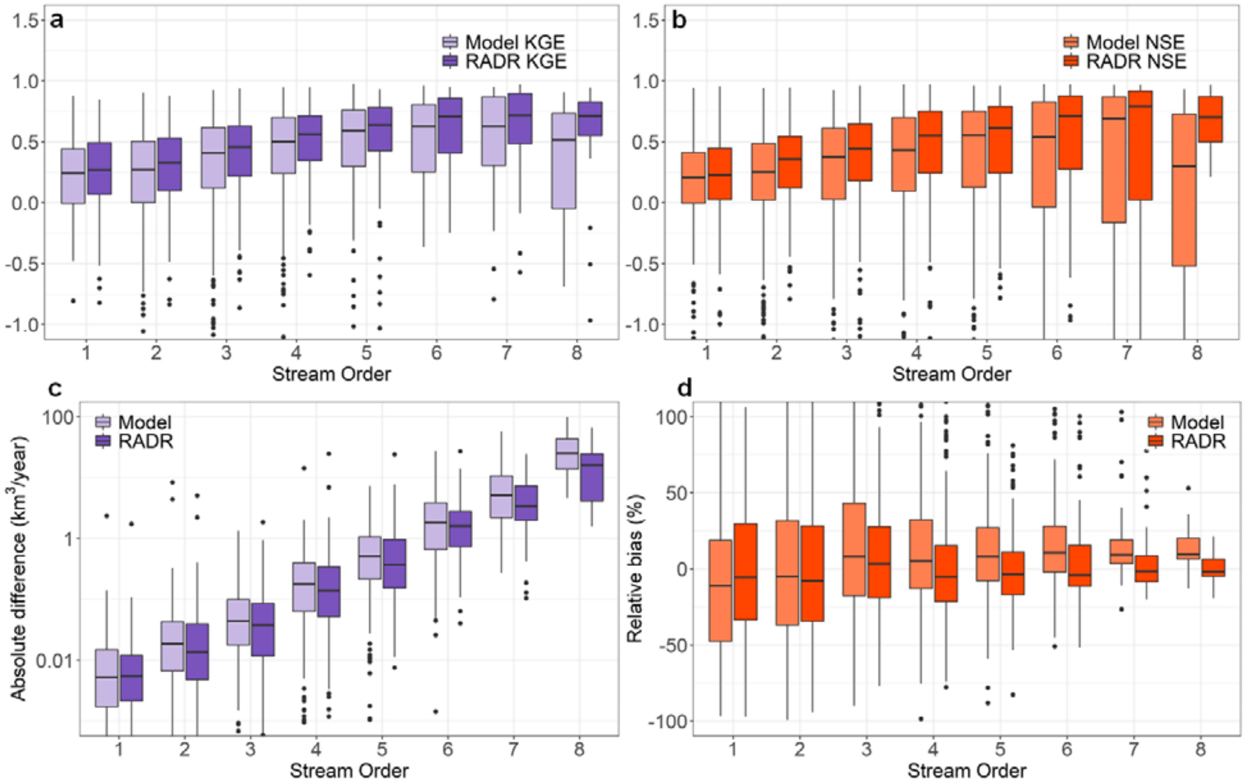

Supplementary Figure 9: Monthly error metrics of RADR and baseline model against all publicly available gauge data from 2,155 gauges (including 1076 monthly gauges and 1079 daily gauges aggregated to monthly), across river sizes (quantified by stream orders). ***a:*** *KGE;* ***b:*** *NSE;* ***c****: Absolute difference ($|Q_{sim} - Q_{obs}|$, km^3/yr);* ***d****: Relative bias (($Q_{sim} - Q_{obs}$)/ $Q_{obs} \times 100$, %). In each boxplot, the highest and lowest points of the whiskers are the maximum and minimum KGE or NSE (excluding the outliers); the top and bottom of the box indicate 75% and 25% values of KGE or NSE; the dark black line in the box is the median of KGE or NSE; black dots are outliers, which are defined as data points that are located outside whiskers of the boxplot (i.e., outside 1.5 times the interquartile range above the upper quartile and below the lower quartile).*

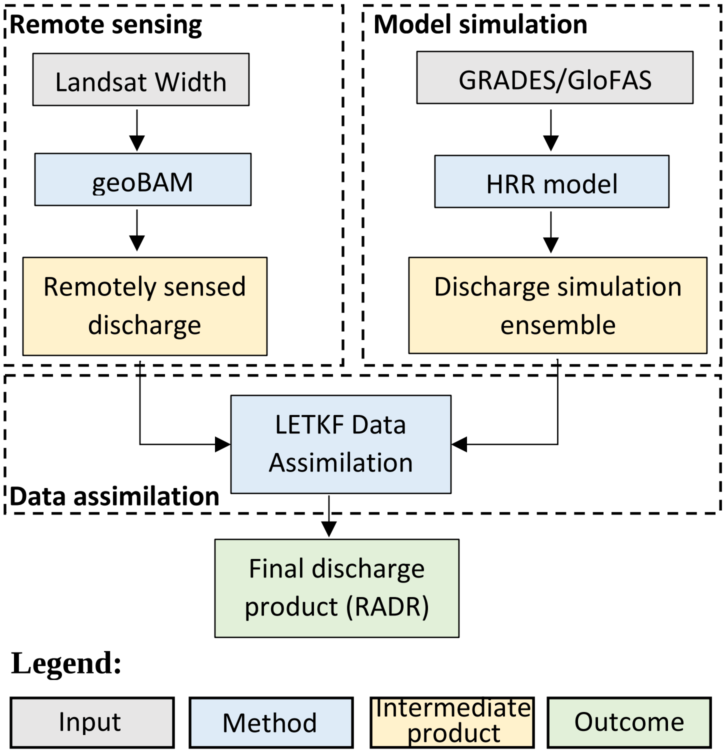

Supplementary Figure 10: Flowchart of the data assimilation process. GRADES[25]: Global Reach-level A priori Discharge Estimates; GloFAS[26]: Global Flood Awareness System; HRR[44]: Hillslope River Routing; geoBAM[19]: Geomorphology-based Bayesian AMHG-Manning algorithm; LETKF[27]: Local Ensemble Transform Kalman Filter; RADR: Remotely-sensed Arctic Discharge Reanalysis.

Supplementary Table 1: Comparison with previous studies regarding the total water export and acceleration in Arctic rivers

Regions	Average annual discharge (km^3/year)	Acceleration rate (km^3/year per year)	Acceleration rate (% per year)	Acceleration significance of RADR	Time period[e]	Sources
Pan-Arctic[a]	5230	3.47	0.07	--	1936-2004	ref[33,34]
	--	5.3	--	--	1950-2004	ref[32]
	5245	--	--	--	1980-2010	ref[29]
	5169	*11.6*	*0.22*	*0.03*	*1984-2018*	*RADR*
EA Composite[b]	1932	2.00	0.10	--	1936-1999	ref[6]
	1796	2.90	0.16	--	1936-2015	ref[29]
	1839	5.34	0.29	--	1964-2000	ref[30]
	1829	10.0	0.55	--	1980-2015	ref[29]
	--	1.87	0.40	--	1981-2011	ref[16]
	1980	*19.7*	*1.00*	*0.01*	*1984-2000*	*RADR*
	2028	*5.07*	*0.25*	*0.13*	*1984-2018*	*RADR*
North America to Arctic Ocean[c]	357	-0.41	-0.11	--	1964-2000	ref[30]
	374	*-1.65*	*-0.34*	*0.19*	*1984-2000*	*RADR*
	378	*0.47*	*0.09*	*0.46*	*1984-2018*	*RADR*
Yukon and Mackenzie	--	0.59	0.23	--	1975-2015	ref[16]
	528	*-2.62*	*-0.50*	*0.48*	*1984-2000*	*RADR*
	522	*-0.35*	*-0.07*	*0.30*	*1984-2018*	*RADR*
HJUBs[d]	717	-2.49	-0.35	--	1964-2000	ref[30]
	714	-2.60	-0.36	--	1964-2000	ref[36]
	764	*0.27*	*0.04*	*0.89*	*1984-2000*	*RADR*
	803	*3.18*	*0.40*	*0.004*	*1984-2018*	*RADR*

[a] Pan-Arctic is the study region of this work, including all rivers draining into the Arctic Ocean, Bering Strait, and the Hudson, James, and Ungava Bays (HJUBs) and not including the Greenland Ice Sheet.

[b] EA composite includes the six greatest Eurasian Arctic rivers: Ob', Yenisey, Lena, Kolyma, Pechora, and Severnaya;

[c] North America to Arctic Ocean are North American rivers excluding the Arctic Archipelago and those draining into the Bering Strait and the HJUBs;

[d] HJUBs includes rivers draining into the Hudson, James, and Ungava Bays;

[e] We also calculated results of RADR for 1984-2000, which is close to the end dates of ref[6,30,31,36].

Supplementary Table 2: Statistics of simulated river discharge and associated trends based on RADR of the 30 largest basins in the pan-Arctic and the Arctic Canadian Archipelago region for 1984-2018.

River Names	Annual mean discharge (km^3/yr)[a]	Uncertainty of annual mean discharge (km^3/yr)[b]	Acceleration rate (%/yr)[c]	Acceleration p-value[c]	Relative bias (%)	Bias (km^3/yr)	Years with in situ daily data during 1984-2018
Yenisey	637	±62	0.118	0.614	0.0	0.2	1984-2018
Lena	577	±56	0.933	0.003	10.3	53.9	1984-2018
Ob’	414	±40	-0.112	0.700	14.1	51.2	1984-2018
Mackenzie	310	±30	0.004	0.953	7.3	21.0	1984-2017
Yukon	212	±20	-0.328	0.116	8.6	16.9	1984-96, 2001-18
Pechora	164	±16	0.134	0.553	-12.0	-22.5	1984-90
Kolyma	130	±13	0.192	0.614	0.5	0.6	1984-94, 1999-2018
Nelson	111	±11	0.743	0.003	5.8	6.1	1987-2016
Severnaya	106	±10	-0.213	0.553	2.0	2.1	1984-2000, 2007-08, 2010-18
Khatanga	102	±10	-0.127	0.790	-2.2	-2.3	1984-1991
Koksoak	65	±6	0.070	0.058	n/a[d]	n/a	n/a
Anadyr	59	±6	-0.076	0.859	5.8	3.3	1984-86,1988-94
Indigirka	58	±6	0.298	0.406	-8.2	-5.2	1984-1996
Pyasina	55	±5	0.084	0.722	n/a	n/a	n/a
La Grande	48	±5	0.096	0.002	n/a	n/a	n/a
Olenek	47	±5	-0.756	0.044	-12.3	-6.6	1991, 1993, 1995-99, 2017-18
Thelon	46	±4	-0.115	0.836	-8.6	-4.3	1984-2018
Yana	39	±4	0.037	0.882	-1.5	-0.6	1984-99,2017-18
Taz	37	±4	0.125	0.236	n/a	n/a	n/a
Moose	36	±4	0.733	0.146	8.0	2.7	1984-2015
Albany	34	±3	0.254	0.657	-19.9	-8.4	1984-2016
Pur	31	±3	-0.101	0.678	-2.8	-0.9	2017-18
Taymyra	28	±3	0.164	0.374	n/a	n/a	n/a
Mezen	26	±2	-0.314	0.459	-6.8	-1.9	1984-99,2017-18
Severn	22	±2	0.420	0.163	15.8	3.0	1984-1994
Hayes	19	±2	0.522	0.123	-15.1	-3.4	1984-2016
Back	19	±2	0.674	0.260	-12.2	-2.6	1984-2010,2012,2014-16
Winisk	17	±2	0.469	0.248	-18.8	-3.9	1984-2015
Anabar	16	±2	0.280	0.022	n/a	n/a	n/a
Churchill	14	±1	0.321	0.343	-33.5	-7.2	1984-2016
Archipelago	269	±26	0.045	0.767	n/a	n/a	n/a

[a] A bias-correction process was applied to basins with gauges near the outlets and then scale up to the outlet reaches based on the drainage areas.
[b] The uncertainty is estimated based on the average absolute relative bias of RADR comapred with gauge observations.
[c] The acceleration rate and p-value are calculated based on using the Mann-Kendall test with a pre-whitened time series.
[d] n/a indicates that there is no in situ daily data during the study period.

Supplementary Table 3: Cross-section arrangement for width extraction

Mean channel width (m)	Cross-section spatial interval (m)
>5000-3000	3000
3000-2000	2000
2000-1000	1000
1000-120	500
120-90	300
